# Chemical profiling of surface water and biota in protected marine harbours impacted by combined sewer overflows

**DOI:** 10.1016/j.envint.2025.109417

**Published:** 2025-04-07

**Authors:** Jasmin Uhlhorn, Keng Tiong Ng, Leon P. Barron, Alex T. Ford, Thomas H. Miller

**Affiliations:** a Centre for Pollution Research & Policy, Department of Life Sciences, Brunel University London, Kingston Lane, Uxbridge UB8 3PH, UK; b MRC Centre for Environment and Health, Environmental Research Group, School of Public Health, Faculty of Medicine, Imperial College London, 86 Wood Lane, London W12 0BZ, UK; c Institute of Marine Sciences, School of Biological Sciences, University of Portsmouth, PO4 9LY, UK

**Keywords:** Occurrence, Pharmaceuticals, Pesticides, Transitional Waters

## Abstract

Few studies exist that focus on contaminants of emerging concern (CECs) in transitional and coastal waterbodies. This study presents chemical profiling of two protected marine harbours on the South coast of the UK sampled in 2022. Across 21 sites, 105 unique compounds were detected (0.05 ng L^-1^ ––1798 ng L^-1^, median: 11 ng L^-1^) in water samples and biota, including 67 pharmaceuticals, 29 pesticides and nine recreational drugs. There were significant differences between campaigns with increased chemical numbers and concentrations that coincided with increased rainfall and combined sewer overflow (CSO) discharges. The comparison with CSO discharges revealed that they were an important source for loading of specific chemicals with concentrations increasing for some cases by three-orders of magnitude. High relative risks were estimated for sites sampled during recorded CSO discharges for five compounds with risk quotients (RQs) ranging from 1.1 up to 9.3, with the highest risk from the neonicotinoid, imidacloprid. To understand the exposure in biota, six species; one macroalgae (*Fucus vesiculosus*) and five fauna (*Hediste diversicolor, Patella vulgate, Crassostrea gigas, Carcinus maenas, Echinogammarus marinus)* were analysed (n = 5/species) at a CSO-impacted site. Between eight to 18 compounds were detected with *Fucus vesiculosus* (seaweed) showing the highest accumulation with mean cumulative burdens reaching up to 343 ± 71 ng g^−1^. Surface water contamination did not correlate with body burdens. Overall, the work highlights the complexity of the chemical space present in a transitional waterbody showing dynamic contamination patterns that are further influenced by tide, rainfall and salinity. CSOs demonstrated an important but compound specific role for CEC input and pulsing into receiving waters.

## 1 Introduction

Anthropogenic contaminants including pharmaceuticals, pesticides, industrial chemicals are prevalent in the environment with contaminants typically found in parts per billion (ppb) to parts per trillion (ppt) range across various compartments including in solid matrices such as sediment (Amos Sibeko et al., 2019; Wilkinson et al., 2018), soils (Gworek et al., 2021; Sabzevari and Hofman, 2022) and sludge (Mejías et al., 2021) as well as water types including wastewater (Khasawneh and Palaniandy, 2021; Rivera-Jaimes et al., 2018) surface water (Galindo-Miranda et al., 2019; Wilkinson et al., 2022) groundwater (Bunting et al., 2021; Lapworth et al., 2012), marine water (Ojemaye and Petrik, 2019) and in organisms inhabiting these spaces (Belenguer et al., 2014; Świacka et al., 2022; Miller et al., 2018). However, chemical monitoring of aquatic environments has often focused on freshwater ecosystems whilst transitional, coastal and marine waters have remained understudied (Branchet et al., 2021). These surface water bodies have changing salinity gradients that could affect chemical fate, and represent unique habitats for a wide range of aquatic fauna and flora where there is limited ecotoxicity data available for these species’ assemblages. Current environmental quality standards are often exceeded in transitional and marine waters with further uncertainty from the limited ecotoxicity information that is extrapolated from freshwater data (Gustavsson et al., 2017; Ghekiere et al., 2013).

Pharmaceuticals encompass a wide range of unique chemicals that are designed to be readily absorbed and elicit a desired physiological effect, which are considered contaminants of emerging concern (CECs). Due to frequent use, these compounds are commonly detected in surface water primarily entering through wastewater treatment plants (WWTPs). Similarly, recreational drugs which can overlap with pharmaceuticals via their misuse may also enter waterbodies through WWTP discharges. Removal efficiencies for these and other micropollutants can vary widely between WWTPs (Fernández-López et al., 2016; Munro et al., 2019; Rapp-Wright et al., 2023) leading to a significant point source for chemical contamination in the aquatic environment. For other contaminants, such as pesticides, input is typically considered from diffuse sources such as leaching and run-off from agricultural applications (Challis et al., 2018). However, more recently it has been shown that some specific pesticides such as companion animal parasiticides (*e.g*. imidacloprid, fipronil) have input from wastewater stemming from domestic use on pets leading to a significant down-the-drain emission pathway (Perkins et al., 2024), as well as from treated animals swimming activity (Yoder et al., 2024). To add to the complexity of source emissions, waterbodies are often further impacted by combined sewer overflows (CSOs) which prevent the capacity of sewer systems being exceeded during periods of rainfall. However, CSOs lead to influent being directly input into surface waters which presents a hazard to aquatic life and public health (Munro et al., 2019; Botturi et al., 2021). Current knowledge gaps surrounding CSOs are related to the volume and composition of spills (Perry et al., 2024) further limiting our understanding for source apportionment (Chrapkiewicz et al., 2024).

The aim of this work was to determine chemical profiles crossing a range of unique and important habitats associated with two harbours (Chichester Harbour and Langstone Harbour), both designated as Sites of Special Scientific Interest (SSSI) for biology that have been impacted by pollution from wastewater (Ford and Ginley, 2024) hence the focus of CECs in the present study. To address the aim, surface water samples were collected from 21 sites impacted by WWTP and CSO discharges and analysed using a targeted quantitative LC-MS/MS method for several classes of CECs (148 analytes). Surface water was collected in March (spring) and November (autumn) where input related to wastewater was expected to be increased from storm events. A qualitative screening method that could tentatively identify an additional 350 contaminants was applied to further characterise the broader chemical space. Finally, biological samples were also analysed from Langstone Harbour in the region which included five different species of fauna (*Hediste diversicolor, Patella vulgate, Crassostrea gigas, Carcinus maenas, Echinogammarus marinus*) and a macroalgae (*Fucus vesiculosus*) to determine the potential for accumulation. To improve our understanding of risk in the marine environment, it is critical that chemical exposure and hazard are better characterised and pathways that drive exposure are identified.

## 2 Materials & methods

### 2.1 Chemicals & Reagents

A total of 148 chemicals were targeted for quantitative analysis in this study by liquid chromatography-tandem mass spectrometry (LC-MS/MS). All non-labelled chemical standards were of analytical purity (≥97 %) and sourced from Merck Life Sciences (Dorset, UK). Additionally, 36 stable isotopically-labelled internal standards (SIL-IS) were of certified analytical purity (≥97 %) and purchased from Merck Life Sciences or QMX (Essex, UK). The full chemical standard list and details are presented in Table S1 of the Supplementary Information (SI). For instrumental analysis, all solvents including methanol (MeOH) and acetonitrile (MeCN) used were of high-performance liquid chromatography (HPLC) grade (or LC-MS grade) sourced from Fisher Scientific (Loughborough, UK). Ultra-pure water was obtained from a Merck Millipore Milli-Q water purification system with a specific resistance of 18.2 MΩ cm or greater. Ammonium formate (HPLC grade), acetic acid (HPLC grade) and formic acid (LC-MS grade) were sourced from Fisher Scientific. All stock solutions were prepared in MeOH or MeCN at 1 mg mL^−1^ and stored in unsilanised amber glass vials (20 mL). Working solutions were prepared from stocks daily in ultra-pure water or MeOH, as required. All solutions were stored at − 20 °C in in amber vials to reduce possible degradation.

### 2.2 Sample location & collection

**Fig. 1:**
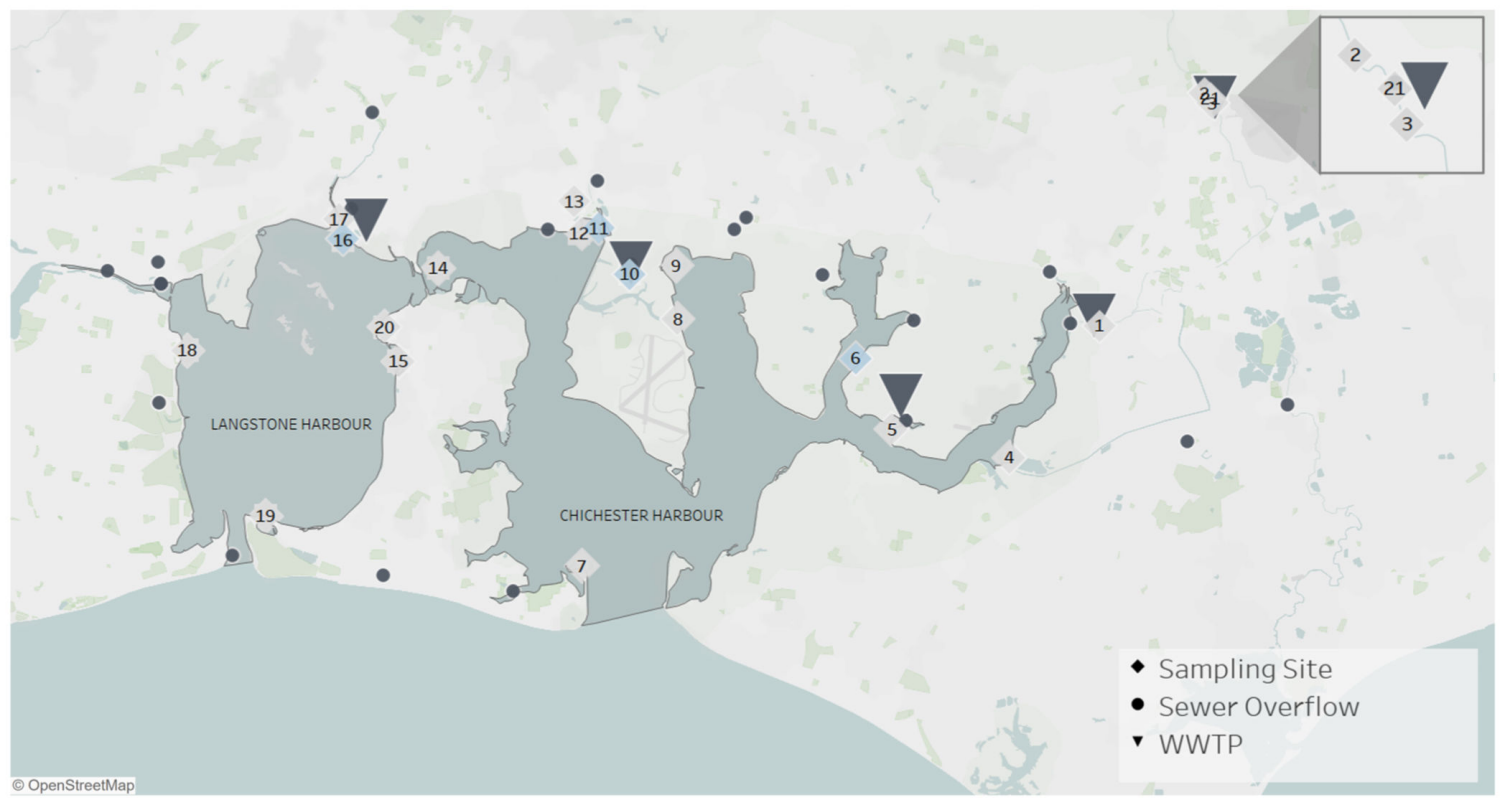
Map showing locations of selected sites (diamonds) for chemical profiling across Langstone and Chichester Harbours in March and November 2022. Blue diamonds indicate the four sites (6, 10, 11, 16) that were re-sampled in November. Triangles represent local WWTPs and circles represent all associated sewer overflow outfalls in the region. (For interpretation of the references to colour in this figure legend, the reader is referred to the web version of this article.)

The study was focused on chemical occurrence in Langstone and Chichester Harbours which have been reported to have declining water quality (Environment Agency, 2024; Natural England, 2021). All sites were selected with assistance from citizen scientists from the Clean Harbour Partnership (CHP) that covered tributaries flowing into the respective harbours and samples from the harbours themselves. The CHP advised on sites that were near known discharge points (WWTP or CSO), that were accessible and used by local water users (*e.g*. sailing clubs). Surface water samples were collected across 21 different sites in the region in March (Fig. 1 & Table S2). Of these 21 sites, four sites were re-sampled in November (Sites 6, 10, 11 and 16) and selected based on the proximity to known WWTP effluent and CSO discharge points in the two harbours and increased rainfall in the region compared to March. Rainfall data for the region was taken from https://environment.data.gov.uk/hydrology/station/125b2167-6fe0-4e51-8976-50a733f3d690.

Additionally, one site (Site 16) was sampled in August 2022 as an *ad hoc* sampling event in response to an observed CSO discharge by members of the CHP. All samples were collected for 5 consecutive days (triplicate) in March, August and November unless otherwise stated (Table S2).

Biota samples (*n* = 30 total, *n* = 5 per species) were collected only in March from two locations in Langstone Harbour and included seaweed (*Fucus vesiculosus*), ragworms (*Hediste diversicolor*), limpets (*Patella vulgata*), oysters (*Crassostrea gigas*), crabs (*Carcinus maenas*) and shrimp (*Echinogammarus marinus*). Surface water samples were collected by members of the CHP in Nalgene bottles (60 mL) following a training session on sampling. Samples were stored in insulated cool bags on ice during collection and then frozen at −20 °C as soon as possible on the same day. After each sampling event the frozen samples were transported to the lab on ice within 6 h and then stored at −20 °C. Eight of the 21 sites were located within 1 km downstream of WWTPs and storm overflow discharge points (Fig. 1 & Fig. S1). All sites were tidally influenced and subsequently all samples were collected on the ebb tide on consecutive days to ensure consistency between sampling intervals and locations. Full specification of sampling dates, times and sites can be found in Table S2.

### 2.3 Sample preparation

For biota, the sample preparation followed methods described previously (Miller et al., 2019; Miller et al., 2021). Both limpets and oysters were de-shelled before extraction but the crab samples were not de-shelled due to the small size (<2 cm in diameter). Briefly, 20 mg of lyophilised solid sample material was weighed into a 2 mL Eppendorf tube. These were then spiked with SIL-IS at 50 ng g^−1^ and extraction was performed using 2 mL of 3:1 MeCN:H_2_O acidified with 0.1 % acetic acid. The sample was vortexed briefly (30 s), then sonicated for 15 min and finally centrifuged for 5 min (4 °C at 14,000 rpm). The supernatant (1.9 mL) was diluted with 50 mL 10 mM ammonium acetate in H_2_O. The pre-concentration and clean-up step was performed using SPE with a Strata® Alumina-N cartridge (1 g, 6 mL; Phenomenex Ltd) and an Oasis HLB cartridge (200 mg, 6 mL, Waters Ltd) configured in series. Cartridges were conditioned with 6 mL methanol followed by 6 mL 10 mM ammonium acetate solution. Liquid sample extracts were subsequently loaded and washed with 1 mL H_2_O. The alumina cartridges were discarded. The HLB cartridges were dried separately for 20 min and then stored at –20 °C until required for analysis. The cartridges were eluted with 5 mL MeOH and dried using a TurboVap (Biotage, Hengoed, UK) at 40 °C until the solvent was completely evaporated. The sample was reconstituted in 0.1 mL mixture of MeOH:MeCN:H_2_O (5:5:90, v/v). The reconstituted sample was filtered using a 0.2 μM centrifuge filter (hydrophilic-PTFE membrane) and transferred to a 0.3 mL glass insert held within a 2 mL autosampler vial with a PTFE/silicone septum cap.

Surface water samples were processed similarly and as described in Miller et al., 2019 and 2021 (Miller et al., 2019; Miller et al., 2021). Water samples were thawed overnight in the fridge at 4 °C and filtered through a 0.2 μm PTFE syringe filter. A 10 mL aliquot from each sample was measured in a volumetric flask. The sample was subsequently loaded onto a conditioned HLB cartridge (conditioned with 6 mL MeOH, 6 mL H_2_O) and washed (1 mL H_2_O). The cartridge was dried and stored as described above. For analysis the cartridges were eluted with 5 mL MeOH and dried. The samples were reconstituted in 300 μL H_2_O:MeCN (95:5 v/v) and transferred to amber glass (non-silanised) screw cap vials.

### 2.4 Instrumental analysis

For biota extracts, the instrumental analysis followed the method published by Egli et al., 2021 (Egli et al., 2021). A LCMS 8060 (Shimadzu Corporation, Kyoto, Japan) was used for targeted quantitative analysis using at least two MRM transitions. Separations were performed on a Shimadzu Nexera X2 ultra high-pressure LC (Shimadzu Corporation, Kyoto, Japan) configured with a short 5.0 × 3.0 mm, 2.7 μm particle size Raptor™ biphenyl cartridge (Thames Restek, Saunderton, UK) housed within an EXP® Direct Connect Holder. Multiple reaction monitoring (MRM) was performed with polarity mode switching, and quadrupoles Q1 and Q3 were set to unit resolution. Full MS conditions are provided in the SI (Table S3). The injection volume was 10 μL and MeCN was used as a wash solvent between injections.

For water samples, A SCIEX Triple Quad™ 7500 (Sciex, Framingham, MA, USA) was used for targeted analysis. Separations were performed on a Sciex ExionLC™ configured with a 100 x 2.1 mm, 3 μm particle size Luna® Omega Polar C_18_ cartridge (Phenomenex, Maccles-field, UK). MRM was performed with polarity mode switching, and quadrupoles Q1 and Q3 were set to unit resolution (SI Table S3). The injection volume was 20 μL and MeCN:MeOH:IPA:Water (1:1:1:1) was used as wash solvent between injections. A flow rate of 0.5 mL min^−1^ was used for all analysis. Mobile phases A (0.1 % formic acid in H_2_O + 5 mM ammonium formate) and B (0.1 % formic acid in MeOH + 5 mM ammonium formate) were used for separation. The full gradient profile and LC conditions are given in the SI (Table S4).

In addition to the quantitative methods described above, a previously validated LC-MS/MS method for drinking water was applied as a targeted qualitative screening method for an additional 350 analytes as the certified reference material was not available (Stahl-Zeng et al., 2020). This method included pharmaceuticals, pesticides, biocides and industrial chemicals. The screening method used the same instrument (SCIEX Triple Quad™ 7500) and conditions described above and given in the SI (Table S4). The screening method applied tentatively identified a compound based on retention time (t_R_) within a ± 0.65 min window and two MRM transitions. The qualitative targeted screening method was applied to the samples collected in November only and prioritised due to expected increases in input from CSO sources.

### 2.5 Quantification and targeted screening

Quantification of surface water samples was performed using pooled matrix-matched calibration curves prepared from 1 − 500 ng L^-1^. Pooling was performed by taking a 10 mL aliquot from each replicate of selected sites across the sampling intervals and combining them. The pooled samples were then measured volumetrically (10 mL) and spiked with the target analytes. Background correction was done using pooled neat samples. Triplicate extraction blanks were included in the analysis. Quantifications were conducted when linearity was acceptable, defined as R^2^ ≥ 0.98 for a linear or quadratic regression. For several analytes the sensitivity of the method saturated the detector response at high calibrant concentrations (>500 ng L^-1^). For these compounds they are reported as > 500 ng L^-1^ as the concentrations could not be reliably estimated from the calibration curves. Analytes were reported as below the lower limit of quantification (LOQ) when the peak had a signal-to-noise (S/N) ratio below 10:1 and the limit of detection (LOD) was defined as peaks with a S/N ratio of ≥ 3:1.

For biota, species-specific matrix-matched calibration curves were prepared by pooling samples into a bulk mass and weighing out 20 mg of dried material to prepare each calibrant. Pooling was done by taking 100 mg of each sample (n = 5) for each species and then mixed using a vortex. Biota samples were spiked with SIL-IS at a concentration of 50 ng g^−1^ dry weight (dw). Pharmaceuticals and recreational drugs were spiked to prepare the calibration curve at concentrations of 10, 25, 50, 100 and 200 ng g^−1^ dw. Isotope-dilution using SIL-IS was used to correct for variability and background correction was performed using data from unspiked, pooled sample extracts. Quantification was considered reliable where linearity was acceptable (R^2^ ≥ 0.98) and measured LC-MS/MS signals were above a S/N of 10. All quantified data in biota is presented on a dw basis.

## 3 Results & discussion

### 3.1 Overview of CEC Occurrence in Surface Water Samples from Langstone and Chichester Harbours

Of 148 target compounds in the quantitative LC-MS method, a total of 105 unique compounds were detected across both sampling periods (339 samples: 67 pharmaceuticals, 29 pesticides and nine recreational drugs). The most frequently detected pharmaceuticals (Fig. S1) included carbamazepine (100 %), tramadol (99.7 %), trimethoprim (98.8 %), venlafaxine (98.5 %), sulfapyridine (96.8 %), sulfamethoxazole (95.3 %), diclofenac (93.5 %), memantine (92.9 %), valsartan (92.6 %), and lidocaine (89.1 %). Of all 67 pharmaceuticals detected, 22 were detected at more than 50 % of the sites. The most frequently detected recreational drugs were nicotine (92.6 %) and ketamine (83.8 %) and the metabolite of cocaine, benzoylecgonine (BZE, 94.7 %) which is in line with previous work concerning recreational drugs in UK surface waters (Miller et al., 2019; Miller et al., 2021; Egli et al., 2021).

Across both harbours, 67 compounds were detected in March and 90 during November. The sampling in spring followed an unusually dry period in the region with three-month cumulative rainfall levels defined as ‘notably low’ (Environment Agency, 2022) and total monthly rainfall in Havant was 32 % lower than compared to the long-term average (LTA) (Fig. S2). In contrast, during autumn there was above average rainfall (Sept-Oct 2022), with November itself receiving more than double the LTA (Fig. S2).

For pesticides, the compounds with higher detection frequencies (>50 %) included fenuron (94.7 %), simazine (82.0 %), propamocarb (79.6 %), clothianidin (68.4 %) and imidacloprid (59.9 %). Fenuron was quantified at an average concentration of 102 ± 99 ng L^-1^ (maximum > 500 ng L^-1^). This chemical has been previously detected in several UK sites reaching up to 169 ng L^-1^ (Miller et al., 2019; Miller et al., 2021; Ng et al., 2020). Considering it has not been approved in agricultural applications since 2009, sources remain unclear and LC-MS/MS data from injections of both mobile phase and method extraction blanks were clean (Fig. S3). It was suggested that it is unlikely to be related to agriculture but may stem from use in other sectors such as building and construction (Ng et al., 2020; ECHA, 2024). Similarly, simazine has also not been approved for more than a decade and was detected across 278 samples. This compound which is a WFD priority substance has been detected in WWTP effluent from rural and urban areas in Ireland but not in receiving waters (Rapp-Wright et al., 2023). In a separate study simazine was also detected across several waterways in London (Egli et al., 2023).

Most of the neonicotinoids, which had partial bans since 2018, were completely banned for all outdoor use on any crops from 2020 (Suchenia, 2022). However, regulations for emergency use have seen approval for the compound thiamethoxam for the past four years to combat Yellows Virus in sugar beet (HM Government, 2021). Despite the restricted use, imidacloprid and clothianidin were quantified up to 63.1 and 35.7 ng L^-1^, respectively. Interestingly, thiamethoxam was not detected at any site but clothianidin is also the major metabolite of thiamethoxam which has been demonstrated in humans, insects and plants (Liu et al., 2018; Nauen et al., 2003; Wrobel et al., 2022) so its presence may be related to the emergency use of thiamethoxam. Moreover, an important source of input for imidacloprid has been reported to be related to its use in pet parasiticides (Perkins et al., 2024; Yoder et al., 2024).

Given that several of these compounds are no longer approved for use for agricultural purposes with 3 substances banned since 2002 (Table S5), it is concerning to find them so widely in these transitional waterbodies at high detection frequencies and at relatively higher concentrations when compared to the range of concentrations measured in this study. The presence may stem from legacy use but for specific compounds (*e.g*. imidacloprid) could be further related to market repositioning following usage restrictions in other sectors (Barron et al., 2024).

### 3.2 Comparison of chemical input between spring and autumn following recorded CSO discharges

For samples collected in March, there were ten analytes detected across all 21 sites including carbamazepine, lidocaine, sulfapyridine, tramadol, trimethoprim, venlafaxine (pharmaceuticals), fenuron (pesticide), BZE, ketamine and nicotine (recreational drugs). The number of detected compounds ranged from 9 to 44 within any single sample. Mean concentrations of chemicals in samples from March ranged from 0.12 ± 0.06 ng L^-1^ (propamocarb) up to 129 ± 108 ng L^-1^ (citalopram), with cumulative mean concentrations for all compounds across all replicates reaching up to 995 ng L^-1^ (Fig. S4).

All but three sites (2, 5, 8) showed consistent measured concentrations with inter-day standard deviation below 100 ng L^-1^ across all measured CECs, suggesting source input was less variable during dry weather when input from diffuse sources (*e.g*. leaching and run-off) and storm events (*i.e*., CSO input) is expected to be minimal. Site 5 showed the largest differences in measured concentrations with cumulative concentrations ranging from 26 ± 14 ng L^-1^ to 1769 ± 493 ng L^-1^ dependent on the day (Fig. S5A). The concentrations were elevated on the first two sampling days and statistically significant compared to the last three sampling days (Mann-Whitney U, *p* = <0.001). *Post hoc* testing using Tukey’s test showed that the second day was significantly different compared to the last three sampling days (*p* = ≤0.01). The sampling location was next to a WWTP (Bosham) outlet and close to CSO discharge points. There are two CSOs for this WWTP which include a settled storm overflow (SSO) and a combined emergency overflow (CEO). However, no recorded discharge events occurred from the Event Duration Monitoring (EDM) during or prior to the sampling period and effluent flow for Bosham WWTP was consistent (1909 ± 30 m^3^ d^-1^) across the sampled days. The data indicates that chemical input increased on the first two sampled days but the reason for the pulse is not clear.

Compared with the pharmaceuticals, the measured concentrations of pesticides were lower with average concentrations ranging from; fenuron (102.1 ng L^-1^), imidacloprid (26.8 ng L^-1^), clothianidin (8.3 ng L^-1^), simazine (0.3 ng L^-1^) and propamocarb (0.1 ng L^-1^). Of these quantified pesticides (Fig. S3), none are currently approved for agricultural use except propamocarb. Furthermore, out of 14 pesticides detected in at least one sample, nine are no longer approved for agricultural use in the UK and EU. The ongoing detection of non-licensed pesticides is similar to a Danish study which measured 17 out of 22 pesticides not approved for use (McKnight et al., 2015). In addition to alternative uses for some pesticides (*e.g*. veterinary use) previous works have highlighted the importance of groundwater in the transport and fate of pesticides in the environment (McKnight et al., 2015; Stuart et al., 2006; McManus et al., 2017) with occurrence of legacy pesticides closely associated with mobility in aquifers (McKnight et al., 2015). Therefore, future work should look at long term persistence and transport through soil-groundwater dynamics for these banned substances.

Four sampling sites (Site 6, 10, 11 & 16) were re-sampled in November 2022 after rainfall had increased for the region and where chemical input was expected to increase (Fig. S2 & 7). These four sites were selected as they were in the harbours and close to known point sources. Chemicals separated by major class across the four sites were significantly different (Mann-Whitney U, *p* = <0.001) when compared between the spring and autumn (Fig. S8). An additional 43 compounds were detected in the autumn that were not previously detected during spring at those sites, which include 27 pharmaceuticals, 14 pesticides, and 2 recreational drugs. During autumn as rainfall increased, both the number of compounds and the concentrations determined were higher compared to the sampling in March and the mean concentrations increased by two-fold in November.

This overall trend was not observed for the pesticides which showed a decline in concentrations which varied by site (Fig. S9 and S10). Pesticides showed a significant decline in concentrations at Site 6 that was driven by fenuron. The trends may be expected here as input of pesticides is typically from diffuse sources rather than point sources associated with the sewerage network (McKnight et al., 2015; Bach et al., 2001; Vryzas et al., 2009; Holvoet et al., 2007). Moreover, pesticide applications for many types of crops in the UK often take place between early Spring to early Autumn. With the low levels of rainfall leading up to March and the collection of samples in November this may further explain the lack of similar trends when compared to pharmaceuticals and recreational drugs. A previous long-term study of pesticide application in Greece (>10 years) demonstrated that the highest concentrations of pesticides were detectable from the first period of rainfall after application (Vryzas et al., 2009). Therefore, although there was increased rainfall in November, the timing of sampling from respective pesticide applications could be an important factor to better understand spatiotemporal trends.

**Fig. 2:**
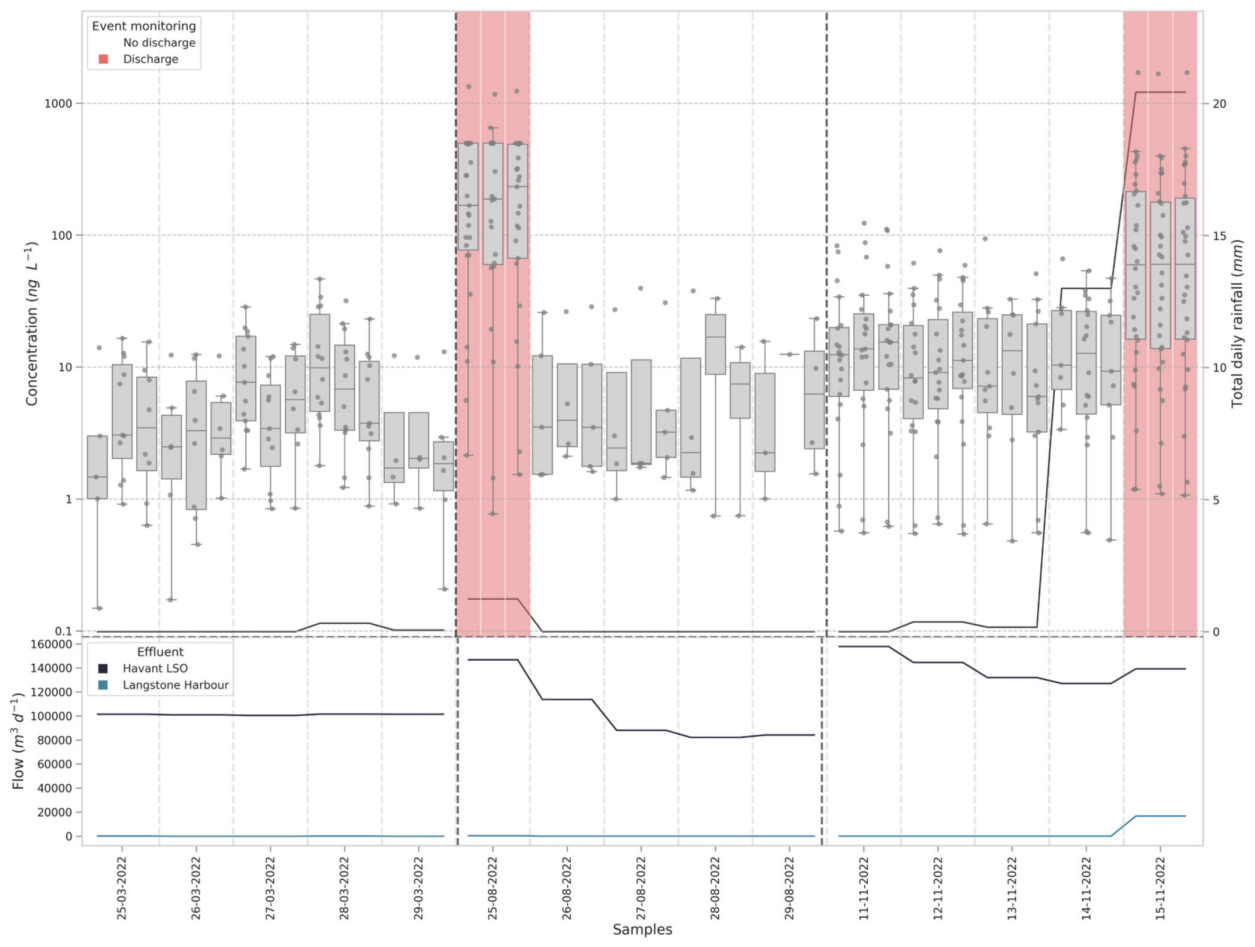
Measured chemical concentrations across consecutive days at Site 16 (near Budds Farm WWTP) in March, August and November 2022 compared to total daily rainfall (top panel). Red panels indicate the dates that a CSO spill event was recorded by the EDM dataset. Effluent outfall flows per day from Budds Farm WWTP, Havant long sea outfall (LSO) discharges to the Solent and the second outfall discharges during heavy rainfall into Langstone Harbour (bottom panel). Line is the median of the data, boxes represent the 25th and 75th percentile, whiskers represent first or third quartile ± 1.5*interquartile range (IQR). (For interpretation of the references to colour in this figure legend, the reader is referred to the web version of this article.)

The higher rainfall in the latter half of 2022 increased the number of CSO events recorded by the EDM data and this was reflected from the measured environmental concentrations in this study. There were additional samples collected in August as a discharge was noted by the CHP and confirmed by EDM data after (Table S6). This reactive sampling took place at Site 16 (near Budds Farm WWTP) which had samples collected in March and November as well. Budds Farm WWTP is the largest facility operated in the study area which serves 366,725 population equivalents (PE), with the other 4 WWTPs in the study area serving between 2,000 – 21,000 PE (Water and Catchment, 2021). Overall, at this site, the mean concentrations compared to March increased up to 43-fold and number of compounds increased by up to 3-fold on the sampling days that coincided with recorded CSO discharges (Fig. 2). Moreover, the increase in chemical concentrations on sampling days that coincided with a recorded CSO discharge were significantly higher than sampling days where no CSO event occurred in both August and November (Mann-Whitney U, *p* = <0.001).

Concentrations peaked on the day of the CSO discharge before returning to baseline concentrations, similar to those recorded during March. The rapid decline in concentrations after the spill event in August is likely related to dilution given the tidal influence of these transitional waters. The concentrations for chemicals that increased relative to the CSO discharge returned to the baseline within 24 h and agrees with observed recovery timeframes from a previous study (Munro et al., 2019). However, the spill in August was short-lived lasting only 5 h (Table S6). The last sampling day for November coincided with a spill event that lasted for 55 h, so there may be a potential for longer spills to have a slower return to baseline concentrations. Whilst duration can be a useful indicator for monitoring CSOs events, the volume of discharges would further increase our understanding of the input and recovery timeframes. Moreover, the pulsing of chemical load into the transitional waters could potentially increase risk for fauna and/or flora that are exposed depending on the recovery period between pulses (Zhao and Newman, 2006; Ashauer et al., 2007; Boxall et al., 2013), where frequent and prolonged pulses may increase sensitivity of organisms.

**Fig. 3:**
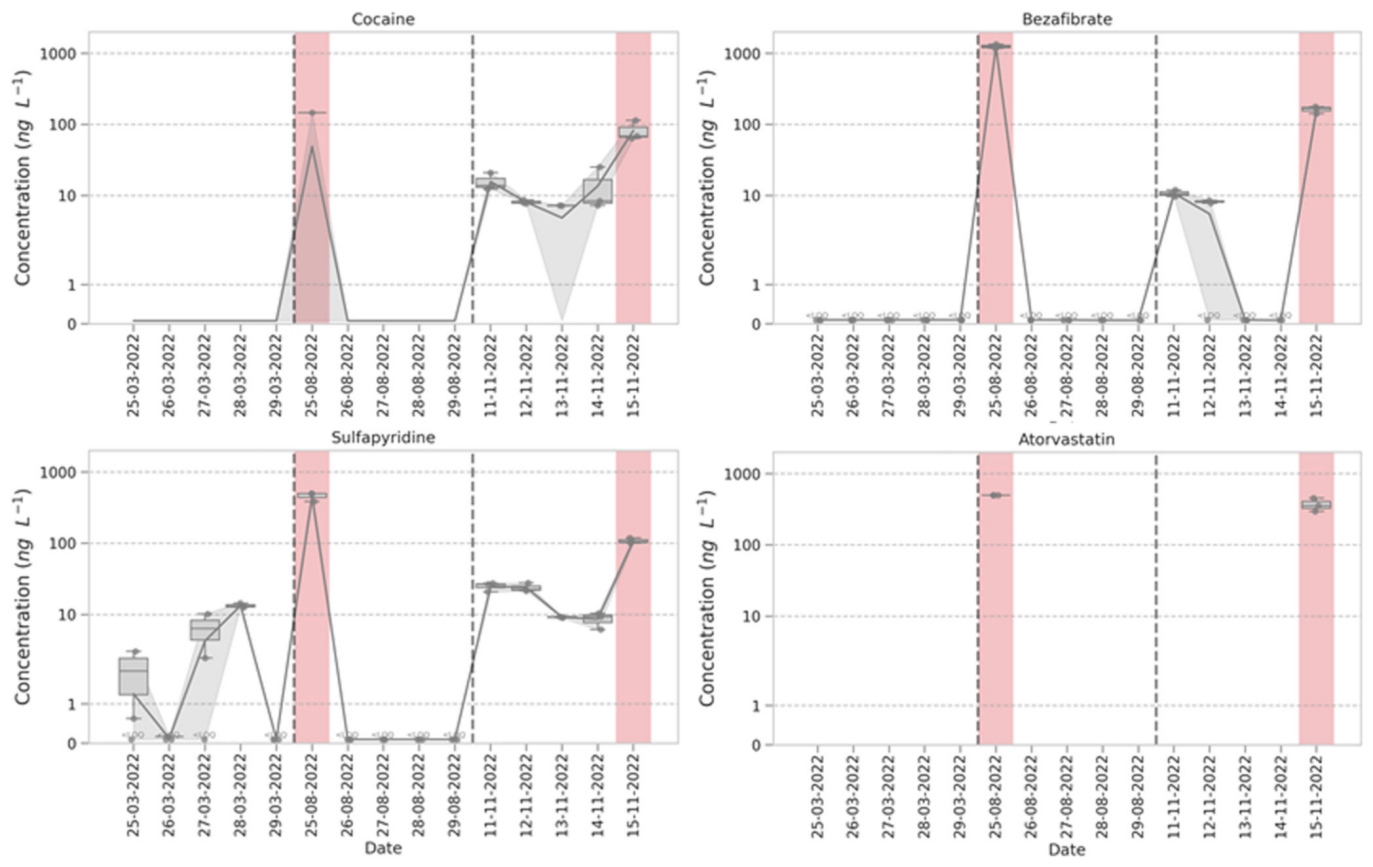
Examples of concentration profiles for individual chemicals; cocaine (top left) bezafibrate (top right), sulfapyridine (bottom left) and atorvastatin (bottom right) measured at Site 16 (near Budds Farm WWTP) in March, August and November 2022. Red panels indicate the date that a CSO spill event was recorded by the event duration monitoring dataset. Line is the median of the data, boxes represent the 25th and 75th percentile, whiskers represent first or third quartile ± 1.5*IQR. (For interpretation of the references to colour in this figure legend, the reader is referred to the web version of this article.)

For Site 16, the average daily concentration of chemicals quantified in March was 6.6 ± 2.7 ng L^-1^, this increased to 63.9 ± 108.6 ng L^-1^ in August and 48.2 ± 59.5 ng L^-1^ in November, showing a minimum increase of 7-fold. On the individual days where a CSO discharge occurred these averages increased further to 281.1 ng L^-1^ (August) and 167.2 ng L^-1^ (November) showing chemical burden increasing by 25 to 43-fold from baseline concentrations in March. Individual compounds showed considerable increases in chemical burden (Fig. 3). For example, valsartan in March was determined at a mean of 11.6 ± 21 ng L^-1^ which increased to 122.4 ± 209 ng L^-1^ August and 245.1 ± 199 ng L^-1^ in November.

**Fig. 4:**
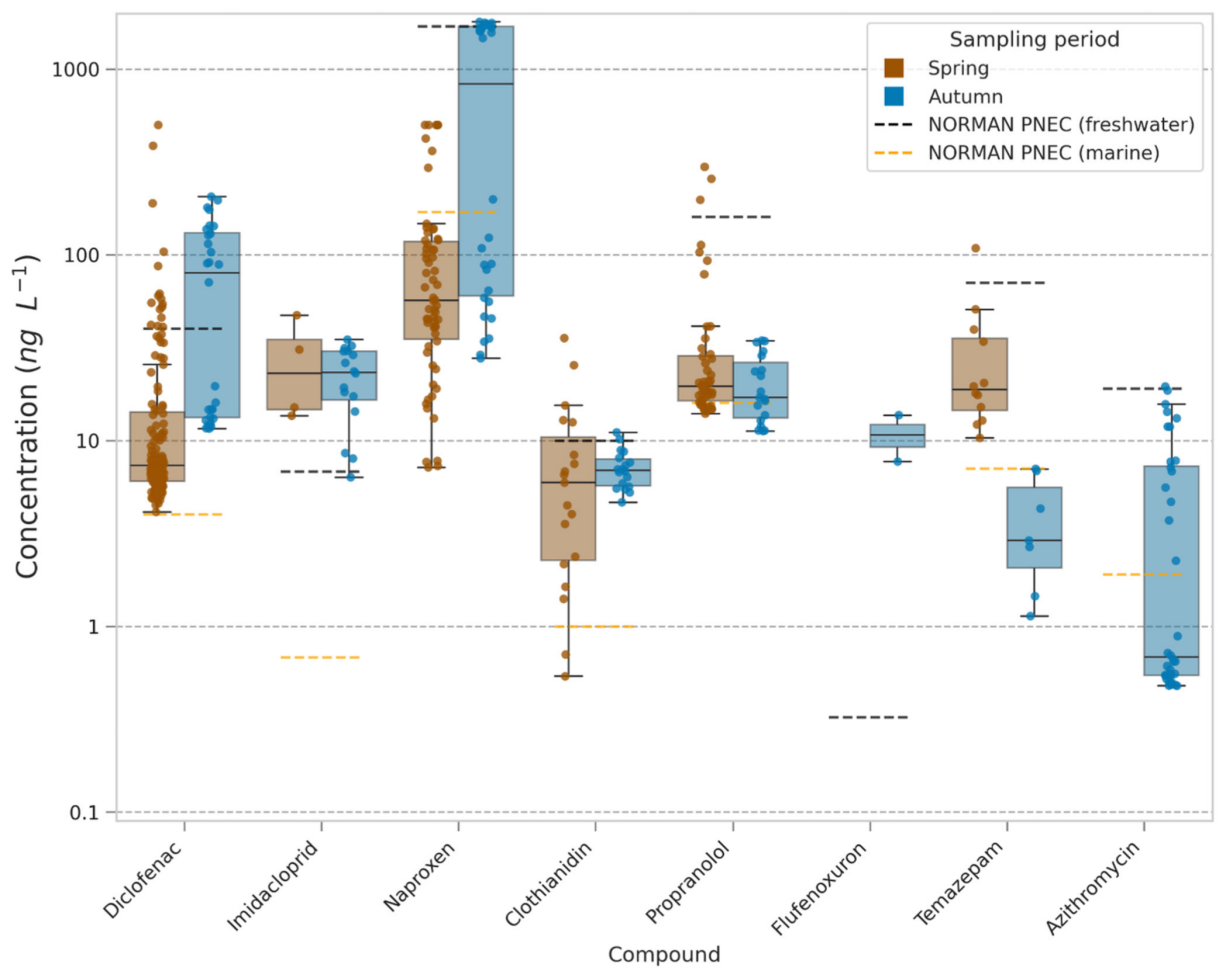
Comparison of quantified chemical measurements between the spring (March) and autumn (November) sampling periods to both freshwater PNECs (black dashed line) and marine PNECs (yellow dashed line). Line is the median of the data, boxes represent the 25th and 75th percentile, whiskers represent first or third quartile ± 1.5*IQR. (For interpretation of the references to colour in this figure legend, the reader is referred to the web version of this article.)

Given the increase in chemical concentrations determined, a comparison to thresholds for predicted no effect concentrations (PNECs) taken from the NORMAN Ecotoxicology Database (Ecotoxicology Database, 2024) (Table S7) was performed for the spring (March) and autumn (November) sampling periods (Fig. 4). The four compounds that exceed freshwater PNECs most frequently were imidacloprid and clothianidin (neonicotinoids) and naproxen and diclofenac (two pharmaceuticals). Quantified concentrations exceeded the lowest PNEC in both sampling periods but were more frequently exceeded during November and potentially related to CSOs. However, there were increased concentrations for multiple compounds in November that stemmed from one site (Site 10).

One antibiotic (azithromycin) exceeded the PNEC in freshwater for one measurement, a further 15 measurements exceeded the marine PNEC value. The lowest PNEC for azithromycin was derived from the cyanobacteria *Microcystis aeruginosa*. For toxicity testing of antibiotics, generally the most sensitive species is microalgae or cyanobacteria in comparison to metazoa such as fish or invertebrates (Fu et al., 2017; Le Page et al., 2017). Moreover, cyanobacteria were equally sensitive when compared to other bacteria including clinically relevant species (Le Page et al., 2017). The risk posed by antibiotics can not only impact ecologically important bacteria and microalgae species but also lead to the spready of antimicrobial resistance (AMR). Previous work has shown that PNECs for surface water (PNEC_SW_) are not always protective of selection pressures that favour AMR, although the PNEC for azithromycin was 10-fold lower than the theoretical PNEC for resistance (PNEC_R(T)_) (Le Page et al., 2017; Bengtsson-Palme and Larsson, 2016). However, knowledge gaps and issues remain for antibiotic risk assessment which are related to the limited number of bacterial species used in testing, lack of testing on marine species and limited data for chronic exposures (Le Page et al., 2017; Sharma et al., 2021).

Imidacloprid exceeded the PNEC in every quantified measurement (apart from in two samples) across both periods with mean risk quotients (RQs) estimated as 3.9 (March) and 3.3 (November) and reaching up to 9.3 across all samples. Other compounds demonstrated RQs reaching up to 7.0 (diclofenac), 3.6 (clothianidin), 1.9 (propranolol) and 1.1 (naproxen). The frequent exceedance of PNECs is a cause for concern and based on these measured environmental concentrations demonstrates that communities are predicted to be negatively affected. Furthermore, the PNECs and RQs are based on individual compounds and do not account for chemicals present as mixtures likely altering their combined risk.

The RQs estimated used PNECs in freshwater as the availability of marine based PNECs is limited and those from the NORMAN Ecotoxicology Database have been estimated by dividing the freshwater PNEC by an assessment factor of 10. Based on these marine PNECs, estimated RQs would increase substantially for the measured chemicals. However, freshwater PNECs are based on either experimentally derived values from specific species or extrapolated from QSAR approaches that limit applicability. There is clear uncertainty regarding the risk for these estuarine and marine environments, and whilst the assessment factor serves as a conservative approach to risk it could potentially underestimate or overestimate the true risk of chemicals measured at these sites.

**Fig. 5:**
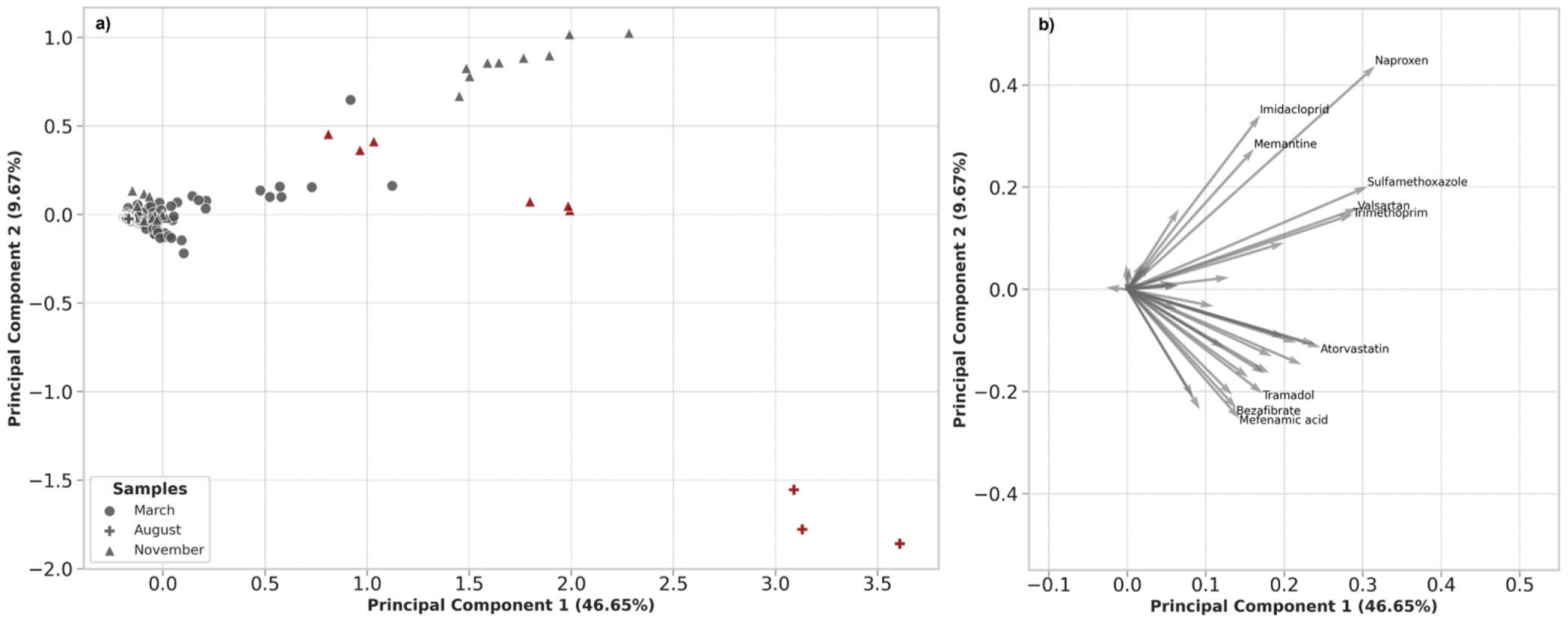
Principal component analysis of quantifiable chemical measurements from all sampling timepoints in the monitoring campaign. (a) Scores plot showing March samples (circles) and November samples (triangles). Red symbols highlight the samples that were collected during recorded CSO events. (b) loading plot showing chemical features with the strongest influence on the clustering. (For interpretation of the references to colour in this figure legend, the reader is referred to the web version of this article.)

A PCA was used to visualise the samples between the different sampling time points across all sites (Fig. 5). The latent variables described 56 % of the data and revealed that generally samples clustered together despite the rainfall increase in November. The exception to this were samples that coincided with recorded CSO spills which separated distinctly from the remaining samples. This indicated that CSOs are an important source for specific compounds in the region. There was not a clear separation between samples collected from sites in March or November indicating that rainfall had less impact on these sites and baseline contamination is similar. However, samples collected from Site 5 clustered more closely with sites influenced by a CSO discharge which is in line with our observations described above. Importantly, all the samples collected from Site 10 (across all consecutive days) in November grouped with samples that were affected by the recorded CSO events (Site 10 and Site 16) despite there being no recorded CSO discharge at this site. The two other sites in the November sampling (Site 6 and Site 11) clustered with the remaining samples in March.

The chemical signatures that drove separation were similar except for samples collected from Site 16 which coincided with a CSO event and clustered distinctly from the remaining samples. Closer inspection of the chemical profiles for Site 16 correlated with known CSO markers including BZE which increased by ~ 20-fold and cocaine which increased by ~ 15-fold (Munro et al., 2019). Caffeine was not part of the quantitative method but was included in the qualitative method where peak areas were noted to increase by two orders of magnitude on the day that coincided with a CSO discharge (for both Site 16 and Site 10). Similarly, other potential CSO markers (Munro et al., 2019) increased such as bezafibrate (<2.4 ng L^-1^ up to 176.7 ng L^-1^) and sulfapyridine (<0.3 ng L^-1^ to > 500 ng L^-1^). We also considered other potential CECs as CSO markers from a previous study that evaluated removal of pharmaceuticals across 45 WWTPs in the UK (Comber et al., 2019). Two compounds (atorvastatin and clarithromycin) showed high removal (median: 85 % and 68 %, respectively) from influent and could potentially be useful as a CSO marker (Comber et al., 2019). Concentrations of atorvastatin were determined < LOD or < LOQ in all samples except at Site 16 and Site 10, with measured concentrations ranging to > 500 ng L^-1^. Clarithromycin followed the same trend remaining < LOQ or < LOD but increasing up to 316 ng L^-1^ from samples coinciding with a recorded CSO discharge or the Site 10 samples.

The clustering may indicate that the WWTP at Site 10 potentially had a lower removal efficiency stemming from lower hydraulic retention time due to increased rainfall and/or groundwater infiltration. However, there had been no rainfall for ~ 48 h before sample collection and effluent flow on the sampled days was significantly lower (Welch’s Test, *p* = <0.05), at 72 % of the average for the month (November average: 20350 m^3^ d^-1^). Alternatively, the data might suggest that there was a CSO discharge across the sampled days at Site 10 due to the increased concentrations of relevant CSO markers which had not been captured by the EDM (Fig. S5D). The issue of CSOs in sewerage systems is well known in the UK, but evidence gaps still exist particularly related to the volume and composition of discharges (Perry et al., 2024). The data presented here suggests pollutant loads from CSO discharges are increased significantly for specific compounds. The risk posed by CSOs discharging into transitional waters is not well established but pulsed exposures in addition to the increased chemical loading as also noted in previous studies (Ford and Ginley, 2024; Phillips et al., 2012; Kay et al., 2017) may exacerbate pressures on these unique ecosystems.

### 3.3 Application of a targeted screening method for tentative identification of chemical presence

**Fig. 6:**
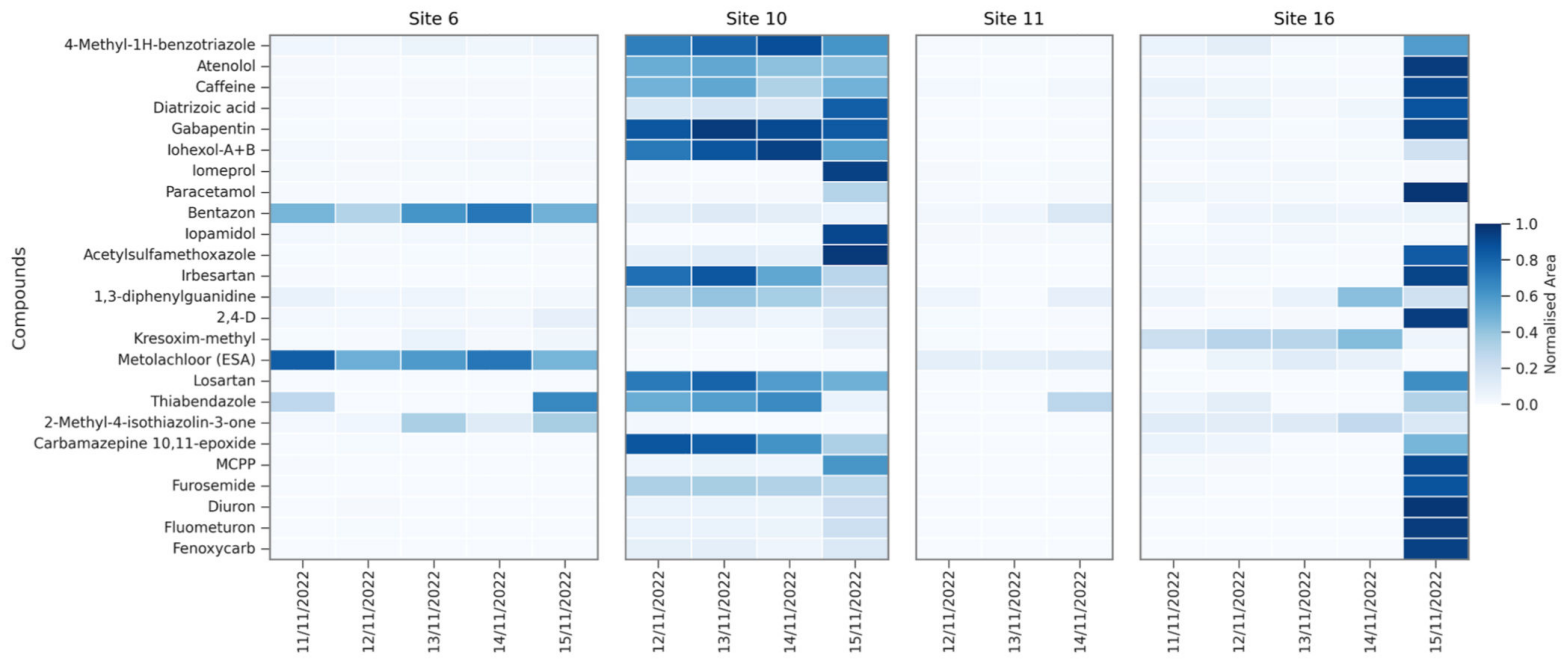
Heatmap showing relative peak areas (normalised by min–max scaling) of the top 25 most frequently detected chemicals tentatively identified using the targeted screening method from daily samples collected from the four sites in November 2022. Tentative identifications were based on the presence of two MRM transitions and a retention time window within ± 0.65 min of expected *t*_R_.

A qualitative targeted screening method was applied to screen the surface water samples collected in November 2022 for additional chemicals where it was expected that chemical input would be increased. The application tentatively identified an additional 50 compounds from these samples which covered pharmaceuticals, biocides, industrial chemicals, pesticides and metabolites (Fig. 6, S11). The most common chemical class tentatively identified was pesticides (n = 23) covering predominantly herbicides (n = 17) or fungicides (n = 5). The most frequently detected (>80 %) compounds were tentatively identified as acetylsulfamethoxazole, bentazone, iopamidol, 4-methyl-1H-benzotriazole, atenolol, caffeine, diatrizoic acid, gabapentin, iohexol, iomeprol, and paracetamol.

The tentative identification of several of these compounds is concerning given that many have biocidal properties and noted to be very toxic in the aquatic environment under ECHA classifications, for example, malachite green, 4-methyl-1H-benzotriazole and methylisothiazolinone. Moreover, of the 23 pesticides tentatively identified, 8 are no longer approved for use in the UK. However, the tentative identification of these compounds is based on the presence of two known MRM transitions and a retention window but are not confirmatory due to the lack of availability of reference materials, so further work would be needed to fully understand the occurrence of substances that are no longer approved for use.

Other compounds of interest included 1,3-diphenylguanidine which is used in rubber production and could indicate the input from tyre-wear particles and road run-off. This compound has been frequently and widely detected across different matrices including soil (Li et al., 2023) drinking water (Marques dos Santos and Snyder, 2023) and surface waters (Johannessen et al., 2021; Zahn et al., 2019; Zhang et al., 2023) but there is still limited understanding of risk from chemicals in tyre-wear which stems from challenges in analytical measurement, lack of routine monitoring, reliability of emission data, mechanisms of toxicity and regulatory approaches (Khan et al., 2024). Contrast agents used in medical imaging were also frequently detected across all sites with 4 compounds having ≥ 90 % detection frequency (diatrizoic acid, iohexol, iomeprol andiopamidol). Two main hospitals are located in the area which are treated by Budd’s Farm WWTP (near Site 16) and Apuldram (Chichester) WWTP (near Site 11). Contrast agents have been again frequently found in the environment as they are stable and persistent but are considered low risk based on hazard assessment (Steger-Hartmann et al., 1999) however some research has suggested that the toxicity of transformation products could be increased in comparison to the precursor compounds (Fabbri et al., 2016; Nowak et al., 2020).

The screening method together with the quantitative method applied in this study highlights the complexity of chemical mixtures found in the environment which furthermore does not account for potential transformation or degradation products. The characterisation of the true chemical space will be important to determine hazard and subsequent environmental risk but will depend on nontarget approaches in chemical monitoring and effect assessment such as effect directed analysis (EDA).

### 3.4 Chemical burden in biological samples collected from Langstone Harbour

**Fig. 7:**
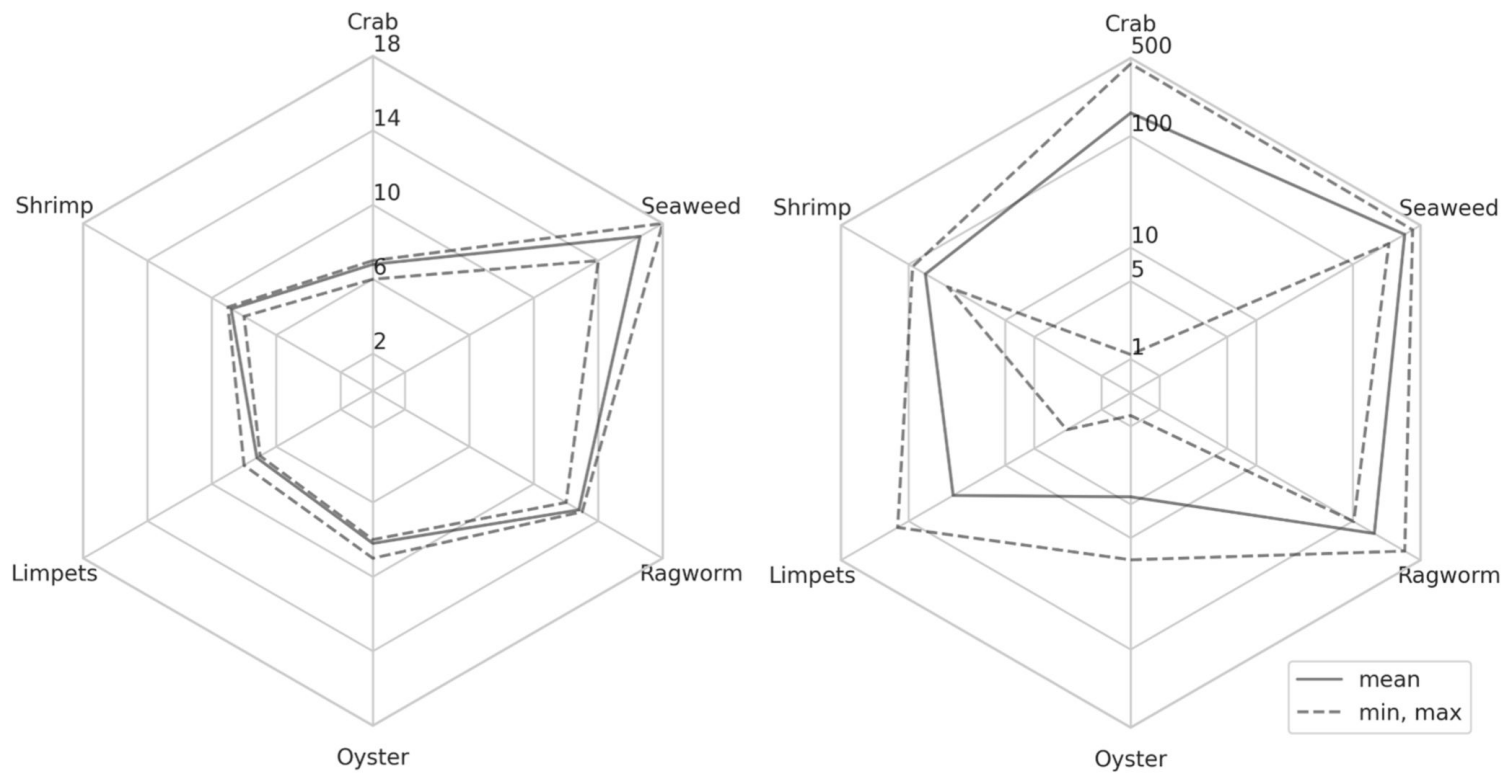
Radar plot of chemicals measured from biota collected from Langstone Harbour March 2022. The mean number of chemicals detected across species with dashed lines representing the minimum and maximum for each (left). The mean cumulative chemical burden (ng g^−1^) determined across species with dashed lines representing the minimum and maximum cumulative chemical burdens for each (right).

Several species of biota were collected from Langstone Harbour during the March sampling period and analysed to better characterise uptake (*i.e*., bioavailability) and potential for accumulation of chemicals in harbour waters. Fauna included crabs, ragworms, shrimp, limpets and oysters, and additionally a common species of seaweed was sampled. Seaweed, ragworms and shrimp were collected by the CSO outfall pipe at Site 16 whereas the oysters, limpets and crabs were collected ~ 2 km south in the main channel, further into Langstone Harbour. The species showed differing levels of chemical burden (Fig. 7, S12). The number of chemicals detected decreased from seaweed (n = 18 compounds) > ragworms (13) > shrimps (10) > oysters (9) > limpets (8) > crabs (8).

The species with the highest body burden corresponded to the seaweed samples which had an average burden of 32.4 ng g^−1^ and an average cumulative burden of 343.2 ng g^−1^. Seaweed has typically been a focus for inorganic analysis with several previous studies showing contamination by metals (Squadrone et al., 2018; Corrias et al., 2020). Only a few studies have looked at organic contaminants in seaweed (Ford and Ginley, 2024; Álvarez-Muñoz et al., 2015; Pacheco-Juárez et al., 2019; Hahn et al., 2022). One study analysed for 6 benzotriazole UV stabilisers (personal care products) that were found to reach up to 115 ng g^−1^ dw (Pacheco-Juárez et al., 2019). A second investigation screened for 35 pharmaceuticals and detected only four compounds that were all below the LOQ (Álvarez-Muñoz et al., 2015) which contrasts with the results observed here. Given that these macroalgae had the highest average burden and high cumulative burdens, this species, which is widespread, may provide a useful bioindicator of chemical exposure in transitional and marine waters.

Individual chemical burden in ragworms averaged 15.7 ± 26.8 ng g^−1^ with an average cumulative burden of 166.4 ± 100.4 ng g^−1^. This was followed by the shrimp which chemical burden averaged 12.9 ± 20.7 ng g^−1^ and an average cumulative burden of 67.1 ± 19.8 ng g^−1^. The relatively higher chemical burden of ragworms compared to other species agrees with our previous study which found that ragworms showed higher body burden compared to a species of shrimp and snail (Miller et al., 2021). The higher body burden in species like ragworms may be related to species traits in that they are an infaunal generalist that burrow into sediments which may potentially be an important exposure route. Sediment as an exposure pathway for emerging contaminants is understudied but previous investigations have shown that this could play an important role for accumulation (Wilkinson et al., 2018; Miller et al., 2021; Celis-Hernandez et al., 2021).

The remaining species showed relatively lower body burdens with average burdens in the order of crab (73.1 ± 110.9 ng g^−1^) > limpets (10.8 ± 8.1 ng g^−1^) > oyster (2.4 ± 2.2 ng g^−1^). The average burden in crab samples was driven by temazepam reaching up to 428.9 ng g^−1^. Removing these values the average chemical burden reduced to 3.4 ng g^−1^. Temazepam was only measured below the LOQ during March but has been previously found in surface waters and detected in wastewater effluents (Rapp-Wright et al., 2023; Lei et al., 2021). Removal efficiencies are not well reported but a previous study indicated a mean removal of 78 % (Wu et al., 2015). A yearlong study in Ireland showed incomplete removal with ~ 35 % remaining in effluent samples from both rural and urban areas (Rapp-Wright et al., 2023). However, concentrations were increased in only 2 out of 5 samples and varied widely in other species (ranging from < LOD to 41.8 ng g^−1^). Bivalves have often been a selected species in monitoring studies (McEneff et al., 2014) as they are filter feeders which filter large volumes of water where the focus has often been on the exposure of contaminants via surface water. Some studies have shown relatively low contamination with individual chemicals typically determined below 10 ng g^−1^ (Álvarez-Muñoz et al., 2015; McEneff et al., 2014; Dodder et al., 2014). However, other studies have shown higher measured concentrations reaching several hundred ng g^−1^ but is likely compound and site specific (Rodil et al., 2019; Pintado-Herrera et al., 2020). Crabs have been less studied and to the author’s knowledge only one study has previously determined organic micropollutants (organophosphates) in crabs that were detected below the LOQs of 1–2 ng g^−1^ (Villegas et al., 2021).

Four compounds were detected in all samples across all species which included cocaine, its metabolite BZE, nicotine and oxazepam. This is similar to our previous work in the UK which detected BZE and cocaine at high frequency (Miller et al., 2019; Miller et al., 2021). However, concentrations were higher in this work with averages of 2.9 ± 4.3 ng g^−1^ and 27.7 ± 23.4 ng g^−1^, respectively. The ratio of cocaine to BZE was 9.5, and the higher level of cocaine agrees with previous studies which showed ratios of > 2.5 in biota (Miller et al., 2019; Miller et al., 2021). At Site 16 (near Budds Farm WWTP) the surface water concentration for these same compounds were typically low, where cocaine was < LOD, BZE was < LOQ (100 % frequency), oxazepam < LOQ (53 % frequency) and nicotine was determined at an average of 11.6 ng L^-1^ (100 % frequency). Measurements compared to surface water samples did not translate well indicating that monitoring studies should incorporate sampling of other compartments within an aquatic system. Without this more holistic overview of exposure our understanding of risk will remain limited.

Individual compound concentrations showed differences between the species sampled. For example, cocaine was determined at higher concentrations in both seaweed and shrimp samples compared to the other species. Ketamine was determined at high concentration in seaweed samples (mean: 198.5 ± 27.8 ng g^−1^) but was comparably low or not detected in other species. Venlafaxine, tramadol and levamisole were also higher in seaweed samples whereas betaxolol, oxazepam and temazepam showed higher concentrations in the ragworms. The species were collected from two sites within Langstone Harbour. The site 2 km further into the harbour (and further away from the CSO outfall) may account for the lower body burden measured in oysters, limpets and crabs where they were sampled highlighting a potential spatial risk for species in close proximity to CSO outfalls. Therefore, the differences observed in occurrence could be related to spatiotemporal exposure history, species specific traits and differences in toxicokinetic rates which has been demonstrated in controlled exposure studies with several macroinvertebrate species to be related to biotransformation and organism morphology (Rubach et al., 2010; Dalhoff et al., 2020).

## 4 Conclusions

A study into chemical pollution in a transitional water body revealed widespread contamination. A total of 105 compounds were detected and an additional 50 compounds tentatively identified from collected surface water samples. A total of 9 pesticides not approved for use in agriculture were confirmed in surface water samples, with some not approved for over a decade. Sources for these remain unclear but could stem from uses beyond agriculture or input from legacy pollution. There were differences observed between the sampling periods in terms of the number of compounds detected and concentration ranges measured, but this was related to sites close to a CSO outfall recorded to have discharged during the sampling. The coincidence with CSOs was clear and we observed a significant increase in chemical exposure with daily averages increasing up to 43-fold related to these discharges. The risk posed by chemicals present in CSO discharges into receiving waters is limited and further studies should investigate the composition, volume and frequency of discharges to better understand the pressure placed on these ecosystems. The biota analysis showed fewer chemicals detected when compared to the surface water samples reflecting bioavailability and accumulation processes. The variation in chemical burden between species is likely reflect exposure history and species traits, highlighting the importance of species selection in biomonitoring studies, and that routine monitoring for example in (inter)national programmes should monitor across species.

Overall, the study demonstrates the complexity of chemical mixtures found in a transitional and coastal water system in the UK. A limited number of studies have focused on these waters but given that they are complex human-environmental systems, it is critical that monitoring should be directed to better understand exposure as the true risk of chemical mixtures is likely to be underestimated especially for marine species which often lack ecotoxicity data.

## Supplementary Material

Supplementary data to this article can be found online at https://doi.org/10.1016/j.envint.2025.109417.

Supplementary Information

Supplementary Information

## Data Availability

Data will be made available on request.

## 5 Data Access Statement

The data underpinning this publication can be accessed from Brunel University London’s data repository, Brunelfigshare here under a CCBY licence: https://doi.org/10.17633/rd.brunel.28513478.
